# Do German university medical centres promote robust and transparent research? A cross-sectional study of institutional policies

**DOI:** 10.1186/s12961-022-00841-2

**Published:** 2022-04-12

**Authors:** M. R. Holst, A. Faust, D. Strech

**Affiliations:** 1 Berlin Institute of Health at Charité – Universitätsmedizin Berlin, QUEST Center for Responsible Research, Charitéplatz 1, 10117 Berlin, Germany; 2 Medizinische Hochschule Hannover, Institute of Ethics, History and Philosophy of Medicine, Carl-Neuberg-Str. 1, 30625 Hannover, Germany

**Keywords:** Science policy, Incentives, Robustness, Transparency, Open science, University medical centre

## Abstract

**Background:**

In light of replication and translational failures, biomedical research practices have recently come under scrutiny. Experts have pointed out that the current incentive structures at research institutions do not sufficiently incentivise researchers to invest in robustness and transparency and instead incentivise them to optimize their fitness in the struggle for publications and grants. This cross-sectional study aimed to describe whether and how relevant policies of university medical centres in Germany support the robust and transparent conduct of research and how prevalent traditional metrics are.

**Methods:**

For 38 German university medical centres, we searched for institutional policies for academic degrees and academic appointments as well as websites for their core facilities and research in general between December 2020 and February 2021. We screened the documents for mentions of indicators of robust and transparent research (study registration; reporting of results; sharing of research data, code and protocols; open access; and measures to increase robustness) and for mentions of more traditional metrics of career progression (number of publications; number and value of awarded grants; impact factors; and authorship order).

**Results:**

While open access was mentioned in 16% of PhD regulations, other indicators of robust and transparent research were mentioned in less than 10% of institutional policies for academic degrees and academic appointments. These indicators were more frequently mentioned on the core facility and general research websites. Institutional policies for academic degrees and academic appointments had frequent mentions of traditional metrics.

**Conclusions:**

References to robust and transparent research practices are, with a few exceptions, generally uncommon in institutional policies at German university medical centres, while traditional criteria for academic promotion and tenure still prevail.

**Supplementary Information:**

The online version contains supplementary material available at 10.1186/s12961-022-00841-2.

## Background

In recent years, the field of biomedicine has seen broad and increasing reflection on its research practices. Various authors have pointed out that flaws in the choice of research questions and in the conduct of biomedical research lead to research waste [1]. These statements have been accompanied by findings that biomedical research often fails to reproduce [2–6], which ultimately hampers the goal of biomedical research, which is translation of findings into medical practice, and ultimately improving healthcare [1].

Concretely, while authors have discussed a possible low base rate of true hypotheses [7], and others have pointed to necessary changes in how research is funded [8] and regulated [9], much of the discussion has focused on the design, conduct, dissemination and reporting of biomedical research. It has been argued that the field fundamentally lacks transparency, with study reports, research protocols or participant data often not publicly accessible, and many research findings not being published at all [10]. If findings are published, they often lack sufficient detail and suffer from selective reporting of outcomes or limitations [12]. In addition, authors have pointed to flaws in biomedical study design and statistical analyses [7, 13, 14]. A recent survey from the Netherlands found that some of these so-called questionable research practices are more prevalent in the biomedical field than in other fields [11].

Several solutions have been proposed to address the flaws in the design, conduct, dissemination and reporting of biomedical research. One of the most widely discussed proposals is the call for more transparent, or “open,” science along all steps of biomedical research. One of these steps is study registration, that is, registering study protocols before data collection, which is supposed to disclose flexibility in data analysis that might lead to false-positive results [15–17]. There have been calls to increase the robustness of science, for example, by asking and supporting researchers in choosing adequately large samples, appropriately randomising participants and performing blinding of subjects, experimenters and outcome assessors [3, 4, 18, 19]. Researchers have been urged to share their data, code and protocols to increase transparency and reproducibility of biomedical research [20], and to report all research results in a timely manner, in line with established reporting guidelines, and ideally without paywalls (open access). This is supposed to tackle prevalent publication bias in which only positive results are reported in journals [21], which distorts the evidence base and thus leads to research waste, for example, by encouraging follow-up studies that would have been considered futile if all research had been reported. To aid in this, new publication formats, namely, preprints and registered reports [22], have been established. All of these procedures are, in the long run, supposed to increase trust in science and lead to more reproducible research [23]. Additionally, more emphasis has been put on actual replication of studies [24], and there have also been calls to abandon [25], redefine [26] or better justify [27] statistical significance thresholds; however, these suggestions have been subject to debate.

To date, the uptake of the aforementioned robust and transparent practices has been slow [28–33]. Many have pointed out that the current incentive structures for researchers do not sufficiently incentivise them to invest in robustness and transparency and instead incentivise them to optimise their fitness in the struggle for publications and grants [34–37]. To receive promotion and ultimately tenure, researchers are evaluated based primarily on how many journal articles (with high impact factors) they publish and how much grant money they secure [35]. The negative influence of the so-called publication pressure on research quality has been shown by mathematical simulations [35, 36] as well as empirical surveys indicating that it is both positively associated with questionable research practices, and negatively associated with responsible research practices [11, 38]. It has been said that all stakeholder groups, including funders and journals, must contribute [9, 12] to an incentive system that actually does reward robust and transparent research practices; in the case of funders, for example, by awarding grants based not only on publication numbers, but on the adoption of open practices and, in the case of publishers, by providing peer review that embraces open practices (allowing peer reviewers to better serve as quality control instances and detect questionable research practices [11]) and not publishing only positive findings, but instead basing editorial decisions just on the soundness of the research. This is, as some studies show, currently not always the case [39, 40].

The role and influence of the research institutions has thus far been less prominently discussed [3]. Since research institutions define the requirements for academic degrees, academic appointments and available intramural funding, their policies and regulations could, and do [11, 38], have a strong impact on researchers’ capability, opportunity and motivation to apply robust and transparent research practices in their work. With regard to university policies, some changes have already been proposed. One of these changes is abandoning the current dysfunctional incentive systems of promotion [35, 36]. Another is an increased focus on transparent practices: the signers of the San Francisco Declaration on Research Assessment (DORA) call for institutions to clearly highlight “that the scientific content of a paper is much more important than publication metrics or the identity of the journal in which it was published” [41]. More specifically, Moher et al. [42] suggest that rewards, incentives and performance metrics at institutions should align with the full dissemination of research, reuse of original datasets and more complete reporting, namely, the sharing of protocols, code and data, as well as preregistration of research (see also the publications by the League of European Research Universities [43] and others [12, 44–47]). Mejlgaard et al. [48] propose that institutions should incentivise making data findable, accessible, interoperable and reusable (FAIR) [49]. Begley et al. [3] suggest similar rules for academic degrees and academic appointments but with regard to the robustness of the research. These authors also demand that the use of reporting guidelines, such as the ARRIVE (Animal Research: Reporting of In Vivo Experiments) guidelines [50] or the CONSORT (Consolidated Standards of Reporting Trials) guidelines [51], be mandated by institutions. Additionally, core facilities such as clinical research units and animal research facilities provide centralised services for the conduct of clinical or animal studies (this includes animal protection and research according to the so-called 3R principles: replace, reduce, refine [52]). These core facilities could have additional influence [53], for example, by recommending that researchers report their results in a timely and nonselective way or by requiring researchers to adhere to established reporting guidelines.

Studying the uptake of the aforementioned recommendations in institutional policies could inform areas for improvement in policy-making at universities. To our knowledge, however, only one study [54] has dealt with this issue, sampling biomedical faculties of 170 universities worldwide and searching criteria for promotion and tenure. The authors report that mentions of traditional criteria of research evaluation were very frequent, while mentions of robust and transparent research practices were rare.

In this cross-sectional study, we aim to describe whether and how relevant policies of university medical centres (UMCs) in Germany support the robust and transparent conduct of research and how prevalent traditional metrics of career progression are. We choose to investigate only German UMCs, as this ensures better comparability of the institutions, since different countries have different regulatory environments (for example, German UMCs are currently in the process of implementing new good scientific practice regulations, mandated by the German Research Foundation [*Deutsche Forschungsgemeinschaft*, [DFG]), different curricula for medical studies and different frameworks for postgraduate degrees. The focus on Germany also allows us to perform in-depth data collection of German-language documents.

## Methods

A detailed methodology is described in our preregistered study protocol, which is available here: https://osf.io/wu69s/ (including a list of protocol amendments and deviations). The following section provides a summary of the methods, which are reported in accordance with the STROBE (Strengthening the Reporting of Observational studies in Epidemiology) [55] guidelines.

### Sampling and search strategy

We obtained a list of all German medical faculties from the website of the German medical faculty council (*Medizinischer Fakultätentag*). For each of the 38 faculties (as of December 2020), we performed a manual search of their websites between 14 December 2020 and 12 February 2021. The search terms and strategy were based on discussions in our full research team after piloting; they have been presented in detail in our protocol. The search was done by the first author (MH), who searched the websites of both the medical faculties and the adjacent university hospitals, looking for the sources presented in Table 1.

**Table 1:** Data sources that were screened in this study

Sources
(1) PhD regulations (for every different type of PhD awarded by the medical faculty)
(2) Habilitation regulations (*habilitation* is an academic degree which is awarded after the PhD, and which involves a second, larger research thesis and additional teaching; it historically was and often still is considered a prerequisite for obtaining tenure or securing third-party funding in Germany [74, 75])
(3) Application forms for tenured professorships
(4) Procedural guidelines for the tenure process (*Berufungsordnungen*)
(5) Websites of clinical research units
(6) Animal research websites, including for animal facilities, 3R centres and animal protection offices
(7) General research websites of the medical faculties or university hospitals

Regarding the PhD and habilitation regulations and the application forms and procedural guidelines for tenure, we saved all related policy documents. Regarding the websites of clinical research units, websites of animal research facilities, 3R centres and animal protection offices, and the general research websites, we first went through each website in detail (including all subpages), saving only those websites and documents that contained any mention of one of the indicators summarised in Table 2. (See Additional file 1: Table S1 for a more fine-grained terminology with subcategories).

**Table 2:** Indicators that were chosen for inclusion in this study

Indicators of robustness and transparency	Traditional metrics
(1) Study registration in publicly accessible registries (e.g. ClinicalTrials.gov, DRKS [German Clinical Trials Register], Open Science Framework, German Animal Study Registry)	(1) Number of publications
(2) Reporting of results	(2) Number and monetary value of awarded grants
(3) Sharing of research data, code or protocol	(3) Impact factor of journals in which research has been published
(4) Open access	(4) Authorship order
(5) Measures to improve robustness	–

We chose both the indicators of robust and transparent research and the traditional metrics of career progression based on their frequent discussion in the literature as either cornerstones of more robust and transparent biomedical research or as incentives leading to the opposite [3, 39, 41, 45, 48]. We also chose them for their consistency with previous research works [54] and publications from our institute [32, 37].

### Data extraction

All documents were imported into qualitative research software (MAXQDA 2020, Release 20.3.0, VERBI GmbH, Germany). We applied deductive content analysis [56]. One rater (MRH) went through all of the documents and coded whether there was any mention of the prespecified indicators of robust and transparent research, as well as the traditional indicators of metrics for career progression. While we searched all documents for the indicators of robust and transparent research, we only searched the PhD and habilitation regulations and application forms and procedural guidelines for tenure for the traditional metrics, as these related specifically to career progression.

If a certain indicator was found, the rater decided whether it was just mentioned (e.g. a university explaining what open access is, or a clinical research unit stating that 60% of clinical trial results were published) or whether that procedure was incentivised/required (e.g. a university specifically requiring a certain impact factor to receive top marks in the PhD or a clinical research unit offering support with summary results reporting of clinical trials). Thus, while we refer to the traditional indicators as “metrics” based on their frequent usage as that, there is no actual difference between indicators and metrics in the sense that they can both incentivise or require behaviour. We based our assessment of incentivised/required on the COM-B model of behaviour change [57], which distinguishes between capability, opportunity and motivation to change behaviour, and lists education, persuasion, incentivisation, coercion, training, restriction, environmental restructuring, modelling and enablement as potential interventions. We defined anything that could increase capability, opportunity or motivation to engage in that behaviour as “incentivised” or “required”.

A second, independent rater (AF) went through the documents of 10 of the 38 UMCs.

## Results

The datasets generated and analysed during the current study are available in a repository on the Open Science Framework (https://osf.io/4pzjg/). The code for calculations of inter-rater reliability, which also includes robustness checks, is available on GitHub (https://github.com/Martin-R-H/umc-policy-review). The inter-rater reliability in our sample of 10 UMCs, measured by Cohen’s kappa, was *κ* = 0.806. Thus, we deemed further double-coding unnecessary.

Overall, the web searches of the 38 German UMCs yielded 339 documents. We found PhD regulations for 37 UMCs (97%), habilitation regulations for 35 UMCs (92%), tenure application forms for 25 UMCs (66%) and procedural guidelines for tenure for 11 UMCs (29%). We found 38 general research websites (100%), 32 websites of clinical research units (84%) and 23 animal research websites (61%; see Table 3). Additional file 1: Table S2 shows numbers for each UMC.

**Table 3:** Number of documents we included for each university and document type

	PhD regulation	Habilitation regulation	Tenure (application form)	Tenure (procedural guidelines)	Website of clinical research unit	Animal research website	General research website
UMCs with document or website found^a^	37 (97%)	35 (92%)	25 (66%)	11 (29%)	32 (84%)	23 (61%)	38 (100%)

^a^For the criteria for promotion and tenure, we counted every UMC with at least one policy found. For the core facility websites, we counted every UMC at which we at least found a website—even if we did not save any documents due to lack of mentions of any of the relevant procedures

The results are presented in detail in Tables 4 and 5, divided by each procedure and each type of document or website. Additional file 1: Tables S3 and S4 provide more detailed data on the subcategories of the indicators of robust and transparent science. Tables 6 and 7 provide example quotes.

**Table 4:** Number of university medical centres that mention indicators of robust and transparent science and traditional indicators of career progression in each of the included sources

	PhD regulation (*n* = 37)		Habilitation regulation (*n* = 35)		Tenure (application form) (*n* = 25)		Tenure (procedural guideline) (*n* = 11)	
	Any mention	Incentivised/required	Any mention	Incentivised/required	Any mention	Incentivised/required	Any mention	Incentivised/required
*Indicators of robust/transparent science*								
Study registration	0% (0)	0% (0)	0% (0)	0% (0)	0% (0)	0% (0)	0% (0)	0% (0)
Reporting of results	8% (3)	3% (1)	3% (1)	3% (1)	0% (0)	0% (0)	0% (0)	0% (0)
Sharing of data/code/protocol	3% (1)	3% (1)	0% (0)	0% (0)	0% (0)	0% (0)	0% (0)	0% (0)
Open access	16% (6)	14% (5)	3% (1)	0% (0)	0% (0)	0% (0)	0% (0)	0% (0)
Robustness	3% (1)	3% (1)	0% (0)	0% (0)	0% (0)	0% (0)	0% (0)	0% (0)
*Traditional indicators of career progression*								
Number of publications	100% (37)	100% (37)	91% (32)	80% (28)	0% (0)	0% (0)	27% (3)	9% (1)
Grant money	0% (0)	0% (0)	11% (4)	3% (1)	84% (21)	0% (0)	27% (3)	9% (1)
Impact factor	16% (6)	14% (5)	63% (24)	54% (19)	72% (18)	0% (0)	0% (0)	0% (0)
Authorship order	97% (36)	97% (36)	80% (28)	80% (28)	68% (17)	0% (0)	0% (0)	0% (0)

The left column for each source indicates any mention of the indicator, and the right column for each source indicates indicators that are incentivised or required

**Table 5:** Number of university medical centres that mention indicators of robust and transparent science in each of the included sources

	Clinical research units (*n* = 32)		Animal research websites (*n* = 23)		General research website (*n* = 38)	
	Any mention	Incentivised/required	Any mention	Incentivised/required	Any mention	Incentivised/required
*Indicators of robust/transparent science*						
Study registration	34% (11)	31% (10)	4% (1)	4% (1)	5% (2)	3% (1)
Reporting of results	9% (3)	3% (1)	4% (1)	0% (0)	21% (8)	11% (4)
Sharing of data/code/protocol	0% (0)	0% (0)	4% (1)	0% (0)	21% (8)	11% (4)
Open access	0% (0)	0% (0)	4% (1)	0% (0)	34% (13)	24% (9)
Robustness	81% (26)	75% (24)	26% (6)	17% (4)	0% (0)	0% (0)

The left column for each source indicates any mention of the indicator, and the right column for each source indicates indicators that are incentivised or required

**Table 6:** Examples of mentions of each practice, divided by policy type (empty sections indicate that no section regarding the metric was found)

	PhD regulation	Habilitation requirement	Tenure (application form)		Tenure (regulation)	
Study registration	–	–	–		–	
Reporting of results	“Doctoral theses must meet high quality standards; after peer review, the written doctoral work should be made accessible to the public and the scientific community as openly as possible through publication.” “Accordingly, the following requirements arise: […] 3. they should lead to a publication in a professional journal or to another kind of publication with a high scientific standard” “According to the recommendations of the DFG (German Research Foundation) […] the following general principles apply to good scientific practice: […] Publication of results”	“The habilitation thesis, or at least its essential parts, are to be published by the habilitated person. The publication should take place within 2 years after awarding of the teaching qualification.”	–		–	
Data/code sharing	“If possible, supervisors should address the following points: […] publication of the full original dataset (e.g., via Figshare, Dryad) of all figures (graphs, tables, in-text data, etc.) in the article.”	–	–		–	
Open access	“If possible, supervisors should address the following points: […] Open Access” “The work can be published: […] 2. As an electronic Open Access publication in the university repository operated by the university library.” “The University Library shall be granted the right to make and distribute further copies of the dissertation as well as to make the dissertation publicly accessible in data networks within the scope of the legal duties of the University Library”	“In the event of publication in accordance with sentence 3 no. 4, the university library shall be granted the right to produce and distribute further copies of the habilitation thesis within the scope of the university library's statutory duties, and to make the habilitation thesis publicly accessible in data networks.”	–		–	
Robustness	“If possible, supervisors should address the following points: […] Reduction of bias by appropriate measures (blinding, randomisation, a priori definition of inclusion and exclusion criteria, etc.), a priori power calculations.”	–	–		–

**Table 7:** Examples for mentions of each practice, divided by policy type (empty sections indicate that no section regarding the metric was found)

	Clinical research units	Animal research facilities	General research website
Study registration	“We offer you the entry of your clinical study both prospectively and retrospectively, i.e., after the study has already started, or support with the entry by yourself.” “The Center for Clinical Trials provides free access to clinicaltrials.gov for scientists of the <institution>. Via this access, investigator-initiated trials from the local area of responsibility can be entered in the registry.” “Since the beginning of this year, all new clinical studies conducted at the University Medical Center have been centrally recorded in the Research Registry University Medicine <institution>” “The analyses will be carried out according to statistical analysis plans that were already designed at the time of study planning”	“The Animal Study Registry provides a platform for pre-registration of an accurate study plan for animal experimental studies prior to the start of experiments, to avoid selective reporting. […] <Institution> 3R sponsors first entries with 500 euros each for research” “The <institution’s> 3Rs toolbox is structured along the lines of the <other institution’s> 6Rs model […], which adds robustness, registration, and reporting to replacement, reduction, and refinement.”	“When should a study project be listed in a public study registry? The International Committee of Medical Journal Editors (ICMJE) requires prospective registration in an ICMJE-recognised registry for all clinical trials to be published by one of the participating journals. The new version of the Declaration of Helsinki also requires this: Article 19 Every clinical trial shall be registered in a publicly accessible database before recruitment of the first subject. The Ethics Committee of the <institution> supports this requirement.” “Recommendation for registration in the DRKS. The International Committee of Medical Journal Editors (ICMJE) requires prospective registration in an ICMJE-recognised registry for all clinical trials to be published by one of the participating journals. The current version of the Declaration of Helsinki also calls for this (Article 35: “Every research project involving human subjects shall be registered in a publicly accessible database before recruitment of the first subject.”). The Ethics Committee of the <institution> supports this call.”
Reporting of results	“ <Institution>: 92% of clinical studies published in EU [European Union] database” “The CRU [clinical research unit] at <institution> offers support with the following tasks, among others: […] Support in writing publications” “In principle, a publication of the results should be aimed at…”	“Fiddle is a tool developed by <institution> to combat publication bias. This ‘match-making’ tool helps researchers to identify alternative ways of publishing information from well-designed experiments, which is often difficult to publish in traditional journals (i.e., null or neutral results, datasets, etc.).”	“As a matter of principle, contributors to research projects are required to actively seek, or at least not refuse, publication of the results.” “As a rule, scientists contribute all results to the scientific discourse. In individual cases, however, there may be reasons not to make results publicly available (in the narrower sense in the form of publications but also in the broader sense via other communication channels); this decision must not depend on third parties.” “Findings that do not support the authors' hypothesis should also be reported.” “Scientific results are to be communicated to the scientific public in the form of publications; the scientific publications are thus—like the scientific observation or the scientific experiment itself—the product of the work of scientists.” “Rules for the publication of results: publication in principle of results obtained with public funds (principle of publicity of basic research), publication also of falsified hypotheses in an appropriate manner and admission of errors (principle of a scientific culture open to error).”
Data/Code sharing	–	“The FAIR Guiding Principles for scientific data management and stewardship state that data must be Findable, Accessible, Interoperable, and Reusable (Wilkinson et al. [49]). These guidelines put specific emphasis on enhancing the ability of machines to automatically find and use the data, in addition to supporting its reuse by individuals.”	“The <city> Open Science programme of <city> has set itself the goal of making the research results and data of research institutions in <city>, which were created with funds from government research funding, freely accessible and easy to find together with other information on science in <city >.” “Scientists at <university> are encouraged to publish and store raw research data that served as the basis for publications, together with the associated materials and information, in recognised open-access subject repositories in accordance with the FAIR principles (Findable, Accessible, Interoperable, Reusable), insofar as this is in conformity with the applicable legal provisions on data protection and copyright and with planned patent applications.” “Self-programmed software is made publicly available with indication of the source code.”
Open access	–	“ <Institutions> will […] establish an “Open access” and “Open Data” culture […].”	“To promote the OA [open access] publishing activities of scientists affiliated with the <university>, the Dean's Office of the Faculty of Medicine has been providing a publication fund from central funds since the beginning of 2020, from which the APCs [article processing charges] for OA publications (original work, review articles) in journals with an impact factor (IF) above 10 are financed.” “Publications in high-ranking open-access journals are supported by the faculty by covering 50% of the costs. Publications can be made free of charge with individual Open Access publishers.” “Therefore, the Presidential Board recommends […] to archive their scientific publications as a matter of principle as pre- or post-print on a subject repository or on the institutional repository of <university>, and/or to publish them in peer-reviewed open-access journals, and to reserve the right to publish or archive their research results electronically in publishing contracts, if possible. In doing so, the freedom of research and teaching is preserved. Discipline-specific practices and rights of use of publishers are to be taken into account in a differentiated manner.” “For a scientific qualification work, e.g., cumulative dissertations or post-doctoral theses, which have appeared in a journal as a published article, permission for second publication must be obtained in any case”
Robustness	“Biometric study planning and analysis includes the following: […] power calculation […] implementation of randomisation” “The biometric consultation hour is an internal service of the CRU for members of the hospital and the medical faculty of the <university>. The biometric consultation hour provides information on questions of study design, sample size planning and the choice of suitable evaluation methods.” “Important tasks of biometrics in clinical trials are power calculation, randomisation” “Online randomisation service”	“The course is aimed at postdocs and scientists who write and/or plan their own animal research applications. In particular, the course will consider what requirements a biometric report must meet in order to satisfy the authorities’ specifications and how this can be achieved. Special attention will be given to power analysis, study design, and sample size calculation.” “Experimental animal science links and information resources: […] G*Power (freeware for the analysis of test power)” “Reduction: The number of test animals is reduced to a minimum. This is achieved by an optimised experimental design, in which it is clarified statistically and methodically in advance how many animals are necessary for an evaluable result.”	–

### Indicators of robust and transparent science

#### Study registration

The issue or relevance of registering studies was not mentioned in any (0%) of the documents regarding academic promotion and tenure. Thirty-four percent of websites of clinical research units mentioned registration, with 31% of those also incentivising or requiring the practice. This appeared mostly in the form of clinical research units offering support with registering clinical studies. Only 4% of animal research websites and 5% of general research websites mentioned registration. The animal facility provided a link to an animal study register, while the two research webpages generally endorsed the practice.

#### Reporting of results

Eight percent of the PhD regulations and 3% of habilitation regulations mentioned the issue of results reporting; these mentions included general requirements that the respective thesis be published. The habilitation regulation also referred to timely publication, asking individuals to publish their thesis no later than 2 years after receiving the degree. Results reporting was also mentioned by 9% of clinical research units, 4% of animal research websites and 21% of general research websites. All mentions expressed general endorsements or highlighted education regarding the publication of all results. One of the clinical research units further offered help with the publication process. The animal research facility that mentioned results reporting provided a tool to identify publication formats that fit the characteristics of the respective datasets. When the general research websites mentioned reporting results, they usually referred to statements in the university’s or the DFG’s good scientific practice guidelines for publishing research.

#### Data/code/protocol sharing

Data, code, or protocol sharing was only mentioned in one PhD regulation (3%). In this mention, supervisors were asked to consider data sharing in the evaluation of the thesis. No habilitation regulations, tenure application forms or procedural guidelines for tenure mentioned this indicator (0%). Likewise, no clinical research unit website mentioned sharing of data/protocols (0%). Four percent of animal research websites and 21% of research websites mentioned data, code or protocol sharing. In the case of the animal facility, the mention was a general introduction to the FAIR principles [49] of data sharing. The general research websites included endorsements of data and code sharing, mostly within the university’s good scientific practice guidelines.

#### Open access

Sixteen percent of PhD regulations and 3% of habilitation requirements mentioned open access. In one PhD regulation, PhD supervisors were asked to also keep in mind whether the work was published with open access. In the other cases, the PhD regulation mentioned that the university library had the right to publish the submitted thesis in a repository (green open access). No clinical research unit (0%) and 4% of animal research websites mentioned open access. In the case of the animal facility, it was a link to an interview in which an “open access culture” was announced. Thirty-four percent of general research websites mentioned open access; these websites either generally recommended open access or referred to the university’s open access publishing funds.

#### Measures to improve robustness

Robustness was mentioned in 3% of PhD regulations but in none (0%) of the habilitation regulations, tenure application forms or procedural guidelines for tenure. Robustness was mentioned by 81% of websites of clinical research units and 26% of the animal research websites. The clinical research units usually offered services to help with power calculations and randomisation (and, in a few cases, blinding). In the case of animal research websites, the mentions pointed to documents recommending power calculation as part of an effort to protect animals, courses on robust animal research and general informational material on these issues. None (0%) of the general research webpages mentioned the issue of robustness.

### Traditional indicators

#### Number of publications

References to publication numbers were made by 100% of PhD regulations and 91% of habilitation regulations. No tenure application documents referred to the number of publications, aside from requirements to provide a complete list of publications. Procedural guidelines for tenure had references to the number of publications in 27% of cases. The PhD regulations and habilitation requirements listed a certain number of publications as a requirement to obtain a PhD or habilitation, respectively.

#### Number and value of grants

None (0%) of the PhD regulations mentioned grant money. Among the habilitation regulations, 11% mentioned grant money, while 84% of the tenure application forms mentioned grant money, in which case there were requirements to provide a complete list of grants awarded. Twenty-seven percent of the procedural guidelines for tenure regulations also mentioned grants. These passages stated that experience with grants was expected or that people were required to provide a list of grants they received.

#### Impact factor

Sixteen percent of the PhD regulations and 63% of the habilitation requirements mentioned an impact factor, with most of them establishing concrete incentives or requirements. These two types of regulations contained passages that asked doctoral students or habilitation candidates to publish in high-impact journals to achieve the highest grade (summa cum laude) or regulations that allowed PhD students to publish only one paper instead of three if that paper was in a sufficiently “good” journal. Tenure application forms mentioned impact factors in 72% of cases, mostly requiring applicants to provide a list of impact factors of each journal they published in. None (0%) of the procedural guidelines for tenure mentioned impact factors.

#### Authorship order

Ninety-seven percent of the PhD regulations mentioned the authorship order, always as an incentive/requirement. The same applied to 80% of habilitation regulations, all of which incentivised or required it. These were regulations requiring PhD students and habilitation candidates to publish a portion of their articles as the first or last author (e.g. a very common regulation for German PhD students is to publish three papers, one of which with first/last authorship). Sixty-eight percent of tenure application forms also mentioned this requirement, noting that applicants should provide a list of publications divided by authorship. None (0%) of the procedural guidelines for tenure had a related section.

## Discussion

In this study, we aimed to assess how and to what extent the 38 German UMCs promote robust and transparent research in their publicly available institutional policies for academic degrees, academic appointments, core facilities and research in general. We also investigated the presence of traditional metrics of researcher evaluation. Our results show that current UMC policies on academic degrees (e.g. PhD regulations) or appointments (e.g. tenure application forms) contain very few (less than 10%) references to our chosen indicators for robust and transparent research, such as study registration, reporting of results, data/code/protocol sharing or measures to improve robustness (e.g. sample size calculation, randomisation, blinding). An exception is open access, which was mentioned in 16% (6 out of 37) PhD regulations, in most cases referring to a repository to which the thesis could be publicly uploaded. In contrast, the number of publications and the authorship order were frequently mentioned in UMC policies on academic degrees and appointments, particularly PhD and habilitation regulations (more than 80%). The majority of application forms for tenure further mentioned impact factors and secured grant money (more than 70%).

The UMCs’ websites for clinical and animal research included more frequent mentions of robust and transparent research, but these differed based on the type of website. Clinical research unit websites frequently mentioned study registration and measures to improve robustness, while animal research websites only had frequent mentions of measures to improve robustness. These mentions were mostly related to sample size calculations and randomization. The general research websites had the most frequent mentions of open access, reporting of results, and data, code or protocol sharing. In most of these cases, these indicators were mentioned in the good scientific practice guidelines. In the case of open access, some websites also featured references to a university-wide open access publishing fund.

Our findings are in line with a similar study that collected data from an international sample [54]. The authors found very frequent mentions of traditional criteria for research evaluation, while mentions of robust and transparent research practices were less frequent than in our study, with none of the documents mentioning publishing in open access mediums, registering research or adhering to reporting guidelines, and only one mentioning data sharing. The results are unsurprising, given recent findings that practices for robust and transparent research are only very slowly becoming more prevalent [30, 32]; however, they stand in stark contrast to the various experts and institutions that have called for institutions to align their promotion criteria with robust and transparent research [3, 41–43, 47, 48, 58, 59]. While we focused exclusively on a full sample of all German UMCs, our approach could also be applied to other countries.

It is important to keep in mind that policies and incentives are constantly changing. As mentioned in the introduction, a major German funder, the DFG, recently reworked their good scientific practice guidelines [60], expecting universities to ratify them in their own good scientific practice guidelines by July 2022. For the first time, these guidelines state that measures to avoid bias in research, such as blinding, should be used and that researchers should document all information and generally should publish all results, including those that do not support the hypothesis. They also recommend open sharing of data and materials in accordance with the FAIR principles and suggest that authors consider alternative publication platforms, such as academic repositories. Some German UMCs might have already changed their internal good scientific practice guidelines by the time the data collection of this study was conducted, which is the reason why we did not explicitly include these guidelines in our web search (we included them, however, if we found them on the general research websites).

One limitation of our study is that the raters were not blinded, which was not possible due to the ability to identify the policies from context. Another limitation is that we only searched for publicly available policies and did not survey relevant representatives of the 38 UMCs personally to identify further policies. For the two types of tenure-related policies in particular, we found relevant policies for only 66% (application forms) and 29% (procedural guidelines) of all UMCs. We refrained from this additional step, however, because the results across the available tenure policies showed a very homogeneous pattern of no mentions (0%) of measures for robust and transparent research, and we assumed that this pattern did not differ across policies that were not publicly available.

While our study focused on reviewing policies for robust and transparent research in policies for academic degrees and academic appointments, as well as their research and core facility websites, there are other ways for institutions to promote these practices. An example is the performance-based allocation of intramural resources, the so-called *Leistungsorientierte Mittelvergabe* (LOM). The LOM might also have a strong influence on researcher behaviour, and it has been proposed that it should be based on transparency of research [61]. Another example would be education on robust and transparent research practices, which has already become a target of reform in Germany. These reforms aim explicitly at training for medical students, who normally do not receive any training in research methodology, to allow them to better understand the evidence base of biomedical research [62–64]. Education aimed at postgraduates might mostly be organised and announced via internal channels of a university and thus not visible for our web search-based methodology. Third, robustness and transparency might be improved by better supervision or better actions against research misconduct, including better whistleblowing systems [48]. Nevertheless, we are convinced that our approach was able to find policies that cover many institutional incentives, especially policies for promotion and tenure, which have a strong influence on researcher behaviour.

Additionally, initiatives for transparent research exist at the federal and national levels (e.g. Projekt DEAL for open access). While universities remain obliged to include these national incentives and policies in their own regulations, future research might focus on these other incentives or policies in the biomedical field.

More generally, there is discussion about how academic institutions—or the academic system in general—need to change to facilitate better research. People have argued that new regulations for open and transparent research might not lead to genuine change for the better, but rather to box-ticking, for example, by arguing that reporting guidelines are not really of help [65] or by showing that study registrations sometimes lack specificity [66]. Additionally, questions have been raised whether assessing individual researchers is the right strategy after all [67]. Criticism has been directed at the general work structures in academia, with some arguing that short-term, non-permanent contracts [68] and a general overweight of third-party funding [69, 70] lead to an unhealthy amount of competition and power imbalances in academia, which in turn facilitate the use of questionable research practices. Research institutions and academia at large are complex systems, with many layers of incentives, and it is yet unclear which measures will lead to a change for the better.

Thus, future research should also address the effects of policies and other institutional activities to increase robust and transparent research practices [71]. Thus far, only a few studies have addressed this. For example, Keyes et al. [72] evaluated the effect of a clinical trial registration and reporting programme, which turned out to be a success. More generally, there is a lack of research on interventions on organisational climate and culture in academia [73].

## Conclusion

In summary, current UMC policies on academic degrees or appointments do not promote procedures for robust and transparent research, especially in terms of policies for academic degrees and academic appointments. In contrast, the number of publications and the authorship order play a dominant role in almost all UMC policies on academic degrees and appointments, and most of the tenure- and appointment-related policies further promote impact factors and grant money secured. This stands in stark contrast to the various experts and institutions that have called for institutions to align their promotion criteria with robust and transparent research.

## Supplementary Information

**Additional file 1: Table S1.** Indicators that were chosen for inclusion in this study (detailed view). **Table S2.** Number of documents we included for each university and document type. **Table S3.** Number of university medical centres that mention indicators of robust and transparent science (fine-grained structure) for career progression in each of the included sources. **Table S4.** Number of university medical centres that mention indicators of robust and transparent science (fine-grained structure) for career progression in each of the included sources.

## Data Availability

The datasets generated and analysed during the current study are available in a repository on the Open Science Framework (https://osf.io/4pzjg/). The code for inter-rater reliability calculations, which also includes robustness checks, is available on GitHub (https://github.com/Martin-R-H/umc-policy-review).

## Abbreviations

ARRIVE: Animal Research: Reporting of In Vivo Experiments
CONSORT: Consolidated Standards of Reporting Trials
DFG: Deutsche Forschungsgemeinschaft (German Research Foundation)
DORA: San Francisco Declaration on Research AssessmentDRKS: Deutsches Register Klinischer Studien (German Clinical Trials Register)
DRKS: Deutsches Register Klinischer Studien (German Clinical Trials Register)
FAIR: Findable, accessible, interoperable and reusable
LOM: Leistungsorientierte Mittelvergabe (performance-based allocation of funding)
PhD: Doctor of philosophy
STROBE: Strengthening the Reporting of Observational Studies in Epidemiology
UMC: University medical centre
3R: Replace, reduce, refine
