# Supplementary material for: Do German university medical centres promote robust and transparent research? A cross-sectional study of institutional policies
Source: Health Res Policy Syst. 2022 Apr 12;20:39. doi: 10.1186/s12961-022-00841-2 (PMC9004041; doi:10.1186/s12961-022-00841-2)
Supplement: Supplementary file 1 — Additional file 1: Table S1. Indicators that were chosen for inclusion in this study (detailed view). Table S2. Number of documents we included for each university and document type. Table S3. Number of university medical centres that mention indicators of robust and transparent science (fine-grained structure) for career progression in each of the included sources. Table S4. Number of university medical centres that mention indicators of robust and transparent science (fine-grained structure) for career progression in each of the included sources. [file 12961_2022_841_MOESM1_ESM.docx]

*Additional Table S1.* Indicators that were chosen for inclusion in this study (detailed view).

| **Sources** |
| --- |
| (1) Study registration in publicly accessible registries (e.g., clinicaltrials.gov, DRKS, Open Science Framework, BfR Animal Study Registry) |
| (1a) in general |
| (1b) prospectively, i.e., before the start of the data collection |
| (1c) adherence to registration guidelines (e.g., WHO Trial Registration Set) |
| (2) reporting of results |
| (2a) in general |
| (2b) in a specified timeframe, e.g., 2 years after the end of the data collection |
| (2c) adherence to reporting guidelines (e.g., CONSORT, STROBE, ARRIVE) |
| (3) sharing of research data, code, or protocol |
| (3a) in general (either data, code, or protocol sharing) |
| (3b) open data |
| (3c) open code |
| (3d) open protocol |
| (4) Open Access |
| (4a) in general |
| (4b) Green Open Access |
| (5) measures to improve robustness |
| (5a) in general (either randomisation, blinding, or power calculation) |
| (5b) randomisation |
| (5c) blinding |
| (5d) sample size calculation |

*Additional Table S2.* Number of documents we included for each university and document type.

| University |  | PhD Regulation | Habilitation Regulation | Tenure (Application Form) | Tenure (Procedural Guidelines) | Website of Clinical Research Unit | Animal Research Website | General Research Website |
| --- | --- | --- | --- | --- | --- | --- | --- | --- |
| Aachen |  | 1 | 1 | 2 | 0 | 5 | 1 | 0 |
| Augsburg |  | 0 | 0 | 1 | 0 | 0 * | 0 * | 0 |
| Berlin |  | 1 | 2 | 1 | 1 | 6 | 7 | 3 |
| Bochum |  | 1 | 1 | 2 | 0 | 0 * | 0 * | 0 |
| Bonn |  | 5 | 2 | 7 | 2 | 2 | 0 * | 0 |
| Dresden |  | 5 | 3 | 2 | 0 | 3 | 0 * | 0 |
| Duisburg |  | 5 | 2 | 2 | 1 | 1 | 0 | 2 |
| Düsseldorf |  | 2 | 1 | 0 | 1 | 3 | 0 | 4 |
| Erlangen |  | 3 | 2 | 1 | 0 | 3 | 0 | 0 |
| Frankfurt |  | 2 | 1 | 1 | 1 | 0 * | 0 | 0 |
| Freiburg |  | 2 | 2 | 3 | 1 | 2 | 0 | 3 |
| Gießen |  | 2 | 1 | 1 | 0 | 1 | 0 * | 1 |
| Göttingen |  | 2 | 2 | 1 | 1 | 1 | 0 | 3 |
| Greifswald |  | 1 | 2 | 0 | 0 | 4 | 0 | 0 |
| Halle-W. |  | 1 | 0 | 1 | 0 | 2 | 0 | 0 |
| Hamburg |  | 3 | 2 | 0 | 1 | 1 | 0 | 3 |
| Hannover |  | 4 | 1 | 1 | 1 | 2 | 1 | 4 |
| Heidelberg |  | 2 | 0 | 2 | 0 | 0 | 0 * | 0 |
| Homburg |  | 2 | 2 | 0 | 0 | 0 * | 0 * | 1 |
| Jena |  | 1 | 3 | 0 | 0 | 1 | 2 | 0 |
| Kiel |  | 2 | 1 | 2 | 0 | 2 | 0 * | 1 |
| Köln |  | 1 | 1 | 1 | 1 | 1 | 0 | 0 |
| Leipzig |  | 2 | 1 | 3 | 0 | 6 | 0 * | 0 |
| Lübeck |  | 1 | 1 | 3 | 0 | 1 | 1 | 2 |
| Magdeburg |  | 7 | 2 | 2 | 1 | 3 | 0 | 0 |
| Mainz |  | 2 | 1 | 0 | 0 | 2 | 0 | 3 |
| Mannheim |  | 2 | 2 | 2 | 0 | 0 * | 0 * | 1 |
| Marburg |  | 5 | 1 | 0 | 0 | 1 | 0 | 0 |
| München LMU |  | 4 | 2 | 2 | 0 | 2 | 0 | 2 |
| München TU |  | 1 | 1 | 0 | 0 | 1 | 0 * | 0 |
| Münster |  | 4 | 2 | 0 | 0 | 3 | 0 | 1 |
| Oldenburg |  | 4 | 2 | 0 | 0 | 0 * | 2 | 4 |
| Regensburg |  | 6 | 1 | 0 | 0 | 3 | 0 * | 0 |
| Rostock |  | 5 | 3 | 1 | 0 | 0 | 0 | 0 |
| Tübingen |  | 2 | 1 | 0 | 0 | 1 | 0 | 1 |
| Ulm |  | 3 | 2 | 2 | 0 | 0 | 0 * | 1 |
| Witten/Herdecke |  | 4 | 2 | 0 | 0 | 1 | 0 * | 1 |
| Würzburg |  | 2 | 1 | 1 | 0 | 2 | 0 * | 0 |
| **UMCs with document or website found ^1^** |  | 37 (97.3%) | 35 (92.1%) | 25 (65.8%) | 11 (29.0%) | 32 (84.2%) | 23 (60.5%) | 38 (100.0%) |

1. For the PhD and Habilitation regulations, the application forms and procedural guidelines for tenure, we counted every UMC with at least one policy found. For the websites, we counted every UMC at which we at least found a website (an asterisk* indicates where not even a website was found) – even if we did not save any documents due to lack of mentions of any of our criteria.

*Additional Table S3a.* Number of university medical centres that mention indicators of robust and transparent science (fine-grained structure) for career progression in each of the included sources. The left column for each source indicates any mention of the indicator, and the right column for each source indicates indicators that are incentivised or required.

|  |  | PhD Regulation  (n=37) | |  | Habilitation Regulation  (n=35) | |  | Tenure (Application Form)  (n=25) | |  | Tenure (Procedural Guideline, n=11) | |
| --- | --- | --- | --- | --- | --- | --- | --- | --- | --- | --- | --- | --- |
|  |  | any mention | incentivised/required |  | any mention | incentivised/required |  | any mention | incentivised/required |  | any mention | incentivised/required |
| **Study Registration** |  | **0% (0)** | **0% (0)** |  | **0% (0)** | **0% (0)** |  | **0% (0)** | **0% (0)** |  | **0% (0)** | **0% (0)** |
| …prospectively |  | 0% (0) | 0% (0) |  | 0% (0) | 0% (0) |  | 0% (0) | 0% (0) |  | 0% (0) | 0% (0) |
| …adherence to guidelines |  | 0% (0) | 0% (0) |  | 0% (0) | 0% (0) |  | 0% (0) | 0% (0) |  | 0% (0) | 0% (0) |
| **Reporting of Results** |  | **8% (3)** | **3% (1)** |  | **3% (1)** | **3% (1)** |  | **0% (0)** | **0% (0)** |  | **0% (0)** | **0% (0)** |
| …specified timeframe |  | 0% (0) | 0% (0) |  | 3% (1) | 3% (1) |  | 0% (0) | 0% (0) |  | 0% (0) | 0% (0) |
| …adherence to guidelines |  | 0% (0) | 0% (0) |  | 0% (0) | 0% (0) |  | 0% (0) | 0% (0) |  | 0% (0) | 0% (0) |
| **Sharing of Data/Code/Protocol** |  | **3% (1)** | **3% (1)** |  | **0% (0)** | **0% (0)** |  | **0% (0)** | **0% (0)** |  | **0% (0)** | **0% (0)** |
| …open data |  | 3% (1) | 3% (1) |  | 0% (0) | 0% (0) |  | 0% (0) | 0% (0) |  | 0% (0) | 0% (0) |
| …open code |  | 0% (0) | 0% (0) |  | 0% (0) | 0% (0) |  | 0% (0) | 0% (0) |  | 0% (0) | 0% (0) |
| …open protocol |  | 0% (0) | 0% (0) |  | 0% (0) | 0% (0) |  | 0% (0) | 0% (0) |  | 0% (0) | 0% (0) |
| **Open Access** |  | **16% (6)** | **14% (5)** |  | **3% (1)** | **0% (0)** |  | **0% (0)** | **0% (0)** |  | **0% (0)** | **0% (0)** |
| …Green OA |  | 14% (5) | 11% (4) |  | 3% (1) | 0% (0) |  | 0% (0) | 0% (0) |  | 0% (0) | 0% (0) |
| **Robustness** |  | **3% (1)** | **3% (1)** |  | **0% (0)** | **0% (0)** |  | **0% (0)** | **0% (0)** |  | **0% (0)** | **0% (0)** |
| …randomisation |  | 3% (1) | 3% (1) |  | 0% (0) | 0% (0) |  | 0% (0) | 0% (0) |  | 0% (0) | 0% (0) |
| …blinding |  | 3% (1) | 3% (1) |  | 0% (0) | 0% (0) |  | 0% (0) | 0% (0) |  | 0% (0) | 0% (0) |
| …sample size calc. |  | 3% (1) | 3% (1) |  | 0% (0) | 0% (0) |  | 0% (0) | 0% (0) |  | 0% (0) | 0% (0) |

*Additional Table S3b.* Number of university medical centres that mention indicators of robust and transparent science (fine-grained structure) for career progression in each of the included sources. The left column for each source indicates any mention of the indicator, and the right column for each source indicates indicators that are incentivised or required.

|  |  | Clinical Research units  (n=32) | |  | Animal Research Facilities  (n=23) | |  | General Research Website (n=38) | |
| --- | --- | --- | --- | --- | --- | --- | --- | --- | --- |
|  |  | any mention | incentivised/required |  | any mention | incentivised/required |  | any mention | incentivised/required |
| **Study Registration** |  | **34% (11)** | **31% (10)** |  | **4% (1)** | **4% (1)** |  | **5% (2)** | **3% (1)** |
| …prospectively |  | 3% (1) | 3% (1) |  | 4% (1) | 4% (1) |  | 5% (2) | 3% (1) |
| …adherence to guidelines |  | 0% (0) | 0% (0) |  | 0% (0) | 0% (0) |  | 0% (0) | 0% (0) |
| **Reporting of Results** |  | **9% (3)** | **3% (1)** |  | **4% (1)** | **0% (0)** |  | **21% (8)** | **11% (4)** |
| …specified timeframe |  | 0% (0) | 0% (0) |  | 0% (0) | 0% (0) |  | 3% (1) | 0% (0) |
| …adherence to guidelines |  | 0% (0) | 0% (0) |  | 0% (0) | 0% (0) |  | 0% (0) | 0% (0) |
| **Sharing of Data/Code/Protocol** |  | **0% (0)** | **0% (0)** |  | **4% (1)** | **0% (0)** |  | **21% (8)** | **11% (4)** |
| …open data |  | 0% (0) | 0% (0) |  | 4% (1) | 0% (0) |  | 21% (8) | 11% (4) |
| …open code |  | 0% (0) | 0% (0) |  | 0% (0) | 0% (0) |  | 5% (2) | 0% (0) |
| …open protocol |  | 0% (0) | 0% (0) |  | 0% (0) | 0% (0) |  | 0% (0) | 0% (0) |
| **Open Access** |  | **0% (0)** | **0% (0)** |  | **4% (1)** | **0% (0)** |  | **34% (13)** | **24% (9)** |
| …Green Open Access |  | 0% (0) | 0% (0) |  | 0% (0) | 0% (0) |  | 13% (5) | 5% (2) |
| **Robustness** |  | **81% (26)** | **75% (24)** |  | **26% (6)** | **17% (4)** |  | **0% (0)** | **0% (0)** |
| …randomisation |  | 66% (21) | 59% (19) |  | 4% (1) | 0% (0) |  | 0% (0) | 0% (0) |
| …blinding |  | 16% (5) | 13% (4) |  | 4% (1) | 0% (0) |  | 0% (0) | 0% (0) |
| …sample size calc. |  | 73% (23) | 66% (21) |  | 26% (6) | 13% (3) |  | 0% (0) | 0% (0) |
